# Low Frequency of MKRN3 and DLK1 Variants in Chinese Children with Central Precocious Puberty

**DOI:** 10.1155/2019/9879367

**Published:** 2019-10-03

**Authors:** Ting Chen, Linqi Chen, Haiying Wu, Rongrong Xie, Fengyun Wang, Xiuli Chen, Hui Sun, Fei Xiao

**Affiliations:** ^1^Department of Endocrinology, Genetics and Metabolism, Children's Hospital of Soochow University, Suzhou, Jiangsu, China; ^2^School of Basic Medicine & Biological Sciences, Medical College of Soochow University, Soochow University, Suzhou, Jiangsu, China

## Abstract

**Background:**

Central precocious puberty (CPP) is defined by gonadotropin-dependent development of secondary sexual characteristics before the age of 8 years in girls and 9 years in boys. *MKRN3* and *DLK1* are two genes, disease-causing variants of which have recently been discovered to cause idiopathic CPP.

**Methods:**

We screened 173 Chinese patients (9 males and 164 females; 9 familial and 164 sporadic) with ICPP and 43 patients (9 males and 34 females; 3 familial and 40 sporadic) with early puberty for variants in *MKRN3*. We also screened 19 patients with ICPP and early puberty for variants of *DLK1* (17 males and 2 females; 5 familial and 14 sporadic).

**Results:**

We identified four novel missense variants of *MKRN3*, c.1138G > A (p.Glu380Lys), c.1420T > A (p.Leu474Met), c.673C > G (p.Leu225Val), and c.1071C > G (p.Ile357Met) in two sporadic cases and three familial cases. According to ACMG standards, two *MKRN3* variant (p.Glu380Lys and p.Ile357Met) are likely pathogenic, and two others are of uncertain significance. We also performed bioinformatic analysis to evaluate the impact of variants on MKRN3 protein structures, which showed that Ile357Met locates at the zinc-binding region (C3HC4 RING finger motif), while Glu380Lys is spatially extremely close to the C3HC4 RING finger, MKRN-specific Cys-His domain, and the third C3H1 zinc-finger motif region. Per Glu380Lys, Glu with negative charges has been changed into Lys with positive charges, which may affect the hydrogen bond formation between amino acids and the stability of the local structure, thus affecting the binding of zinc iron to MKRN3 protein. Besides, we did not identify any variants of *DLK1* gene in our patients.

**Conclusions:**

In this study, we report four novel *MKRN3* variants in patients with ICPP. Moreover, we did not find any variants of *DLK1* gene. Variants of *MKRN3* are relatively uncommon in Chinese ICPP patients.

## 1. Introduction

Central precocious puberty (CPP) is defined pathophysiologically by the premature activation of the hypothalamic-pituitary-gonadal axis and clinically by the development of secondary sexual characteristics before the age of 8 years in girls and 9 years in boys [1]. CPP can be either idiopathic (ICPP) or secondary to CNS lesions. ICPP is much more common compared to secondary CPP, and one-third of ICPP was reported to be familial [2]. Early puberty is usually defined by the age of pubertal onset between 8 and 9 years old in girls and between 9 and 10 years old in boys [3].

**Figure 1:**
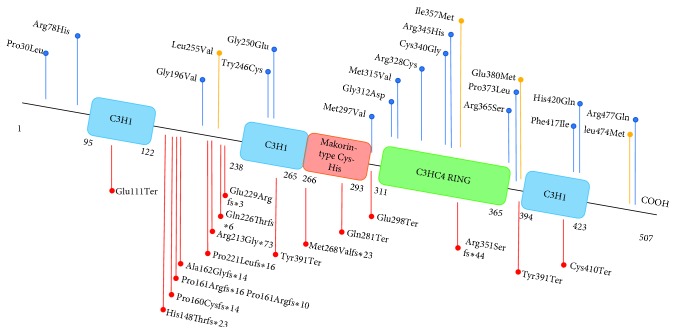
Location of MKRN3 variants identified in patients with central precocious puberty. Blue boxes, zinc-finger domains; red box, makorin-type Cys-His motif; green box, C3HC4 RING finger motif. Red arrows indicate the locations of all reported variants which will cause nonsense or frameshift variants. Blue arrows indicate the locations of all reported missense variants, and yellow arrows indicate missense variants we found in the present study.

To date, only deleterious variants in four genes have been reported as causes of CPP [4–7], including *KISS1*, *KISS1R*, *MKRN3*, and *DLK1*. *KISS1* and *KISS1R* gene defects caused CPP is extremely rare. Besides, the polymorphisms of *KISS1* gene have also been associated with CPP [8, 9]. In 2013, through a series of familial aggregation studies, Abreu et al. discovered that pathogenic variants of the gene encoding makorin RING finger protein 3 (MKRN3) would cause CPP [6]. Since then, about 33 pathogenic variants in *MKRN3* gene have been discovered in CPP patients (Figure 1) [10, 11]. Accumulating evidences showed that pathogenic variants in *MKRN3* are the most frequent genetic cause of familial ICPP [10]. In 2017, Dauber et al. identified a genetic defect in *DLK1* gene, which encodes delta-like 1 homolog, associating with familial ICPP [7]. *MKRN3* and *DLK1* both locate within imprinted loci which were previously revealed to be associated with human puberty initiation through large genome-wide studies [12].

In this study, we screened *MKRN3* and *DLK1* variation in a group of Chinese patients with either ICPP or early puberty, in order to explore the frequency of variants of both genes in Chinese patients.

## 2. Material and Methods

### 2.1. Subjects

The patients reported in this study attended the Endocrinology, Genetics, and Metabolism Department of Children's Hospital of Soochow University (Suzhou, China) between September 1, 2015, and May 31, 2018. A total of 173 patients (9 males and 164 females; 9 familial and 164 sporadic) with ICPP and 43 patients (9 males and 34 females; 3 familial and 40 sporadic) with early puberty were enrolled in this study. All the patients involved in this study are Chinese. Patients with CPP secondary to central nervous system pathology (i.e., tumors or nonspecific cerebral anomalies associated with CPP) were excluded from the study.

Informed consent was obtained from the patients and family members included in this study, following procedures specified by the Ethics Committee of Children's Hospital of Soochow University.

### 2.2. Study Design

All the participants underwent clinical examination, blood sampling, bone age, and brain MRI. The patients had been diagnosed with ICPP (pubertal onset before age 8 in girls and before age 9 in boys) or early puberty (pubertal onset in girls ≥8 years but <9 years old or pubertal onset in boys ≥9 years but <10 years old) on the basis of the appearance of pubertal signs (breast budding in girls and testis enlargement in boys), increased growth velocity, accelerated bone maturation, increased uterine and ovarian volume, and elevated LH response in a standard GnRH stimulation test (LH peak >5 IU/L; LH peak/FSH peak ratio >0.6) [13]. Testis enlargement is defined as testicular volume larger than 4 ml measured by using Prader orchidometer. Blood samples for genetic analysis were obtained from the patients and their family members. All the participants were screened for variants in *MKRN3*. Among the *MKRN3* negative patients, 19 samples (17 males and 2 females; 5 familial and 14 sporadic) were screened for variants in *DLK1* gene.

### 2.3. Biochemistry

Blood samples were drawn from an antecubital vein between 8:00 and 10:00 AM and stored at −20°C until analyses. GnRH stimulation tests were carried out as previously described [13]. Serum LH, FSH, estradiol, and testosterone levels were measured as previously described [13].

### 2.4. Variant Analysis

Genomic DNA was extracted from peripheral blood leukocytes from all patients following standard procedures. The exons of *MKRN3* (NM_005664) and *DLK1* (NM_003836.6) were amplified by PCR followed by automated sequencing of the products. If any variants were found in the probands, Sanger sequencing would also be performed in his or her parents. The primer sequences are listed in Supplemental Table 1. PCR was performed in an ABI 9700 Thermal Cycler under standard conditions. After purification using a QIAquick PCR Purification Kit (Qiagen), the products were directly sequenced onto an Applied Biosystems 3730xl automated sequencer (Life Technologies Corporation Carlsbad, CA, USA). Sequence comparisons and analyses were performed using the Phred-Phrap-Consed program.

### 2.5. Sequence Retrieval, Homology Modeling, and In Silico Mutagenesis

The sequence information of *MKRN3* was obtained from Uniprot database (http://www.uniprot.org, UniProtKB ID: Q13064). The division of domains and classification information were also from this database. We adopted the online server I-TASSER [14] for *ab initio* modeling. We then received the initial overall structure of the wild-type MKRN3 for further analysis. Based on the obtained structure, we polished the domains whose local conformations were available, including three C3H1 domains and one C3HC4 RING finger domain. Firstly, three temples (PDB ID: 1Z6U, 2Y43, and 1RGO) were retrieved via BLAST/PSI-BLAST. Furthermore, we polished the aforementioned four domains based on the initial structure using MODELLER V9.19 [15] platform. Finally, the further modification of the loop region in this structure was carried out, and we obtained the final 3D structure of MKRN3. We applied the VMD1.9.4 [16] platform for the 3D structure visualization and analysis.

## 3. Results

### 3.1. Sanger Sequencing and Familial Segregation Analysis

**Figure 2:**
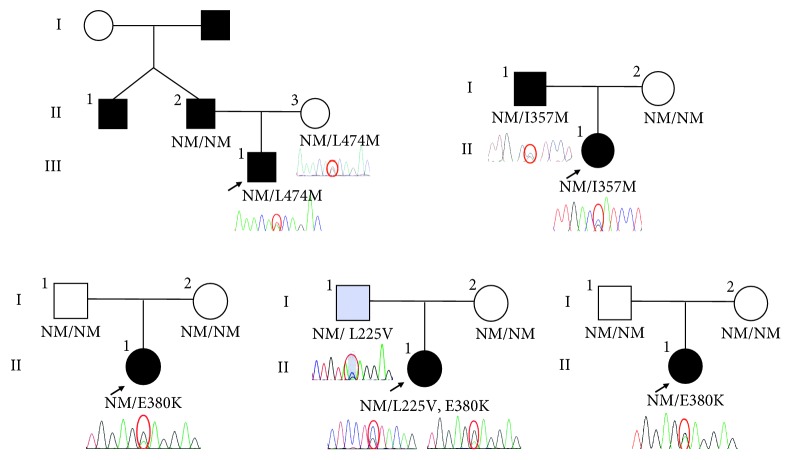
Pedigrees of patients with MKRN3 variants. Arrow, proband; square, male; circle, female; black symbols, subjects with idiopathic central precocious puberty; grey symbols, subjects with early puberty; WT, wild type. Roman numerals indicate the generation. Arabic numerals indicate the patients in each family.

Sanger sequencing was used to identify variants of *MKRN3* genes of 173 ICPP patients and 43 early puberty patients. Four novel missense heterozygous variants (c.1138G > A (p.Glu380Lys), c.1420T > A (p.Leu474Met), c.673C > G (p.Leu225Val), and c.1071C > G (p.Ile357Met)) and 2 synonymous heterozygous variants (c.336G > A (p.Gly112GLy) and c.870T > C (p.Ile290Ile)) were detected in ICPP patients (Figure 2). All the 4 novel variants were found neither in ExAC nor in 1000G. Besides, these 4 variants were also not found in our local population database. A paternal mode of inheritance was observed in two familial cases with variants p.I357M (p.Ile357Met) and p.L225V (p.Leu225Val), respectively. A *de novo* missense variant E380K (p.Glu380Lys) was found in 2 sporadic and 1 familial cases of ICPP. The variant p.L474M (p.Leu474Met) was found inherited maternally; therefore, it is unlikely to be pathogenic. One synonymous heterozygous variant was detected in early puberty patients (c.396T > A (p.Val132Val)). One polymorphism (c. 663C > T (p.Pro221Pro)) was found in 81 out of 216 patients, 8 of them were homozygous and others were heterozygous.

Variants of *DLK1* gene were investigated in 19 ICPP or early puberty patients; 5 of them were familial and others were sporadic. Four heterozygous polymorphisms were found: (c.564T > C (p.I188I) in 18 out of 19 patients, c.310G > A (p.V104M) in 2 out of 19 patients, c.699T > C (p.C233C) in 2 out of 19 patients, and c.404 + 221C > T in 7 out of 19 patients).

### 3.2. Clinical and Biochemical Findings

Clinical and laboratory characteristics of patients studied are shown in Table 1. Clinical and hormonal data of patients with *MKRN3* variants are shown in Supplemental Table 2. All ICPP patients were treated by GnRH analogs (either triptorelin or Leuprorelin).

### 3.3. In Silico Structural Modeling and Analysis of the Variants

**Figure 3:**
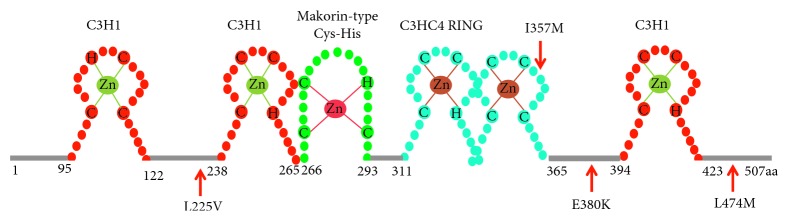
MKRN3 protein structure and variants identified in patients with central precocious puberty. Zn, zinc; H, histidine; C, cysteine. The three C3H1 zinc-finger motifs are shown in red, the C3HC4 RING finger motif is in blue, and the makorin-type Cys-His domain is shown in green. The numbers correspond to the amino acid positions in the protein. Red variant labels and arrows indicate the location of the missense variants.

**Figure 4:**
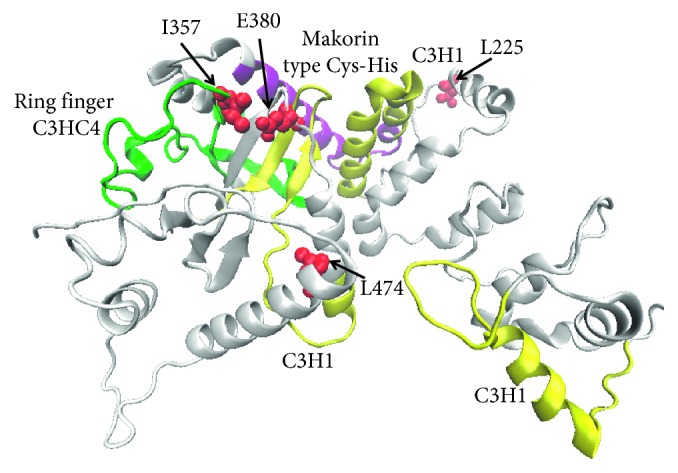
Native structure of the produced MKRN3 protein using *ab initio* modeling. The three C3H1 zinc-finger motifs are shown in yellow, the C3HC4 RING finger motif is in green, and the MKRN-specific Cys-His domain is shown in purple. The four missense variant residues are highlighted with red spheres.

In the present study, we built the 3D structure of MKRN3 and analyzed the situations of the four clinically discovered nonsynonymous variants in the monolithic structure. Moreover, we also predicted the possible molecular mechanisms by which these variants lead to functional impact on the MKRN3 protein. As shown in Figure 3, we found that the protein was composed of five zinc-binding sites, including three C3H1 zinc domains, one makorin-specific Cys-His zinc domain with MKRN family characteristic, and one C3HC4 RING finger domain. From this sequence model chart, we can easily find that I357 (Ile357) locates in the C3HC4 RING finger domain, while other variants L225 (Leu225), E380 (Glu380), and L474 (Leu474)) do not localize in any known zinc-binding region at the sequence level. To figure out the functional effect of these variants, we built 3D structure of protein MKRN3 using modeling software. From the structure diagram (Figure 4), we found that I357 and E380 are spatially extremely close to each other and both locate near the C3HC4 RING finger, MKRN-specific Cys-His domain, and the third C3H1 zinc-finger motif region. Meanwhile, both E380 and L474 are close to the third C3H1 zinc-finger domain spatially. Also, L225 residue is closed to the second C3H1 domain and makorin-specific Cys-His domain.

## 4. Discussion

In the present study, we identified four novel *MKRN3* missense variants in three familial and two sporadic cases. According to the ACMG guideline, the variants E380K and I357M were classified as likely pathogenic, and the variants L474M and L225V were classified as variants of uncertain significance (VUS). No pathogenic variants were found in *DLK1* in our patients.

*MKRN3* locates in the critical region of Prader–Willi syndrome (PWS) and is maternally imprinted. Normally, only the paternal allele is expressed, and the maternal allele is methylated. Therefore, theoretically, CPP can be caused while pathogenic *MKRN3* variants come from the patient's father or when there is maternal uniparental disomy or chromosomal translocations. The *MKRN3* defects are relatively common in European and Latin American CPP patients [10], especially in familial [17] and male [18] ICPP patients. However, *MKRN3* variants caused ICPP are not so common in eastern Asian, according to the results of the present study and previous publications [19, 20].

MKRN3 protein belongs to the makorin protein family, which is characterized by combination of zinc-fingers. The Structure of MKRN3 contains two copies of a C3H1 motif in the N-terminal, followed by a makorin-type Cys-His domain, a C3HC4 RING zinc-finger, and a final C3H1 motif (Figure 3) [6]. Up to date, approximately 33 variants of *MKRN3* gene have been reported, among which 16 are missense mutation and 17 are null mutation. No hotspot region in *MKRN3* gene has been discovered, but half of the premature termination variants locate between the two N-terminal C3H1 zinc-finger motifs. Some missense variants were reported in more than one family. About half of the missense variants reported locate outside the functional domains. Since no functional studies are available, all these reported missense variants should be interpreted with caution. According to previous reports, frameshift or nonsense variants are likely to cause more severe phenotypes when compared to missense variants.

In the present study, we identified four novel variants, which were not found in ExAC, 1000G, or our local database. The variant E380K is *de novo* and predicted to be deleterious by multiple *in silico* tools. Besides, the patient's phenotypes and family history fit MKRN3 defect-caused CPP. The variant I357M is located in critical zinc-finger region and predicted to be pathogenic by several *in silico* tools. Moreover, the variant I357M is inherited from the proband's father with CPP, which fits the specific inheritance pattern of MKRN3 mutation. The variant L225V is also inherited from the proband's father, who had early puberty instead of CPP. *MKRN3* mutations may manifest less severe in males than in females, which was also reported in previous cases [21]. However, since L225V is predicted to be benign by multiple *in silico* tools, the pathogenicity of this variant is still in doubt. The variant L474M is also VUS since it is inherited from proband's mother instead of father.

**Figure 5:**
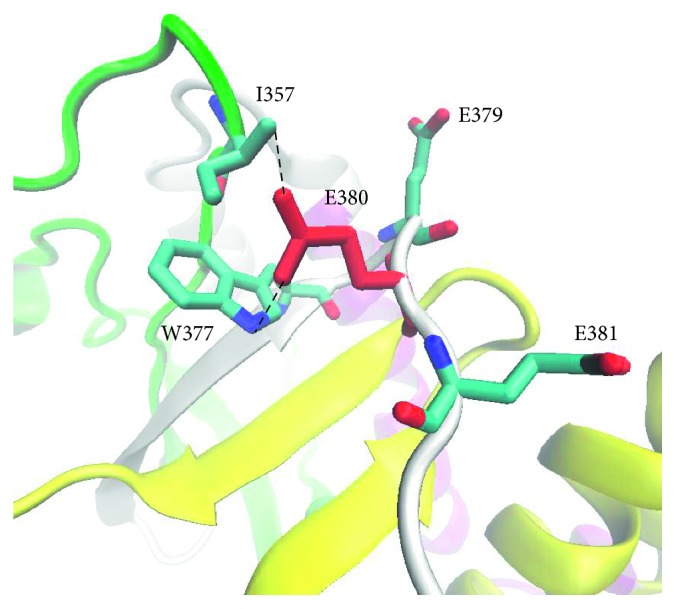
Interaction between E380 and surrounded residues.

Structural analysis reveals that the four variants discovered in the present study are located in different structural regions, which might have various functional effects on MKRN3 during biological processes. The change of Glu to Lys in E380K was suggested to have a notable effect on the function. Since the negatively charged amino acid glutamate is replaced by the positively charged amino acid lysine, the local physicochemical properties of the protein were completely changed, and the interactions with the surrounding amino acids may be altered (Figure 5). Considering the short distance between the sidechains, the native E380 may establish hydrogen bonds with the residues I357 and W377. However, after the mutation, the positively charged lysine will cause disruption of the two pairs of hydrogen bonds and will tend to form hydrogen bonds with E379 or E381 nearby, which in turn might affect the local structure stability, especially the stability of the zinc-binding region. It is highly possible that the local conformational distortions may directly influence the binding of ions. Structurally, the L225V, I357M, and L474M variants are all replacements between hydrophobic amino acids, which only alter the size of the side-chain groups. There are no significant differences in the interaction with surrounding amino acids. But considering the structures, as to the variants of I357M and L474M, the sulfur atom in the side-chain of the mutant methionine might interact with the Zn ion in the zinc-finger domain and change the original binding state between Zn ion and cysteine or interact with the cysteine in the protein, thereby influencing its original function.

Similar to *MKRN3*, *DLK1* gene also locates in a maternal imprinting region located in 14q32, which is originally reported as a critical region of Temple syndrome. *DLK1* encodes a transmembrane protein which is important for adipose tissue homeostasis and neurogenesis. The first case of *DLK1* variant-caused CPP was just reported in 2017 [7]. According to the limited reports, *DLK1* variant may not be a common genetic cause of CPP. In the current study, we have not found any pathogenic variants of *DLK1* gene in our patients [7, 22].

Our study has several limitations. Firstly, the sample size was relatively small, and further studies with larger sample size are needed to confirm our findings. Secondly, we did not sequence *KISS1* and *KISS1R* gene in our patients. Although *KISS1* and *KISS1R* gene-caused CPP is extremely rare, there is still a possibility that the CPP in our patients are caused by defects of these 2 genes. Besides, the sample size of *DLK1* gene sequencing is too small to reveal the real prevalence of these gene mutations in CPP patients. Lastly, since we only explored the pathogenicity of novel variants via ACMG guidelines and bioinformatic approaches, further functional studies are still needed to confirm the results of bioinformatic predictions.

In conclusion, we found 4 novel missense variants of *MKRN3* and no variant of *DLK1* in Chinese ICPP patients. This study suggested that the prevalence of *MKRN3* variants might be low in Chinese ICPP patients. Multicenter studies with larger sample sizes are still needed to clarify the exact prevalence of *MKRN3* and *DLK1* gene defects-caused ICPP in Chinese population.

## Figures and Tables

**Table 1:** Clinical and biochemical features of the enrolled patients.

	CPP patients		Early puberty patients	
	Boys	Girls	Boys	Girls
Patients (*n*)	9	164	9	34
Age at diagnosis (years)	8.5 (7.8–8.9)	7.4 (2.4–7.9)	9.4 (9.1–9.9)	8.2 (8.1–8.8)
BMI SD	0.2 (−0.5–2.0)	0.4 (−0.2–1.7)	1.2 (−0.2–2.8)	0.4 (−1.4–4.8)
Height SD	1.2 (−1.8–3.2)	0.6 (0.2–3.4)	1.1 (−2.1–2.8)	0.7 (−0.8–3.1)
Bone age (years)	11.5 (10.8–12.6)	9.3 (4.0–11.5)	12.6 (11.7–13.4)	10.6 (9.9–12.2)
Basal LH (UI/L)	1.05 (0.15–5.8)	0.94 (0.07–2.31)	1.89 (0.89–6.18)	1.02 (0.16–6.32)
Peak LH (UI/L)	18.7 (8.8–27.5)	9.8 (5.8–97.1)	22.6 (13.2–29.0)	11.1 (8.5–65.7)
Peak LH/FSH ratio	1.8 (0.7–2.5)	1.1 (0.6–2.68)	2.6 (1.1–3.8)	1.3 (0.7–3.2)
E2 for girls (pg/ml)		28.3 (16.8–64.2)		48.9 (19.2–59.6)
T for boys (ng/dl)	85.4 (45.6–185.8)		111.4 (49.5–215.3)	

Data are shown in median and range. BMI, body mass index; SD, standard deviation; LH, luteinizing hormone; FSH, follicle-stimulating hormone; E2, estradiol; T, testosterone.

## Data Availability

The clinical, genetic, and bioinformatic data used to support the findings of this study are included within the article.

## Supplementary Materials

Supplementary Materials Table 1: primer sequences of MKRN3 and DLK1 genes. Table 2: clinical characteristics of patients with MKRN3 mutations. TS, Tanner stage; BA, bone age; FSHb, basal FSH; LHb, basal LH; LHp, peak LH; UL, uterine length; TV, testicle volume.
