# Supplementary material for: Low Frequency of MKRN3 and DLK1 Variants in Chinese Children with Central Precocious Puberty
Source: Int J Endocrinol. 2019 Oct 3;2019:9879367. doi: 10.1155/2019/9879367 (PMC6794979; doi:10.1155/2019/9879367)
Supplement: Supplementary Materials — Table 1: primer sequences of MKRN3 and DLK1 genes. Table 2: clinical characteristics of patients with MKRN3 mutations. TS, Tanner stage; BA, bone age; FSHb, basal FSH; LHb, basal LH; LHp, peak LH; UL, uterine length; TV, testicle volume. [file 9879367.f1.docx]

**Table 1 Primer sequences of *MKRN3* and *DLK1* genes.**

| Gene | Forward primer sequence (5'->3') | Reverse primer sequence (3'->5') |
| --- | --- | --- |
| MKRN3-2 | CCGGAAGTAGGTAGGAACACAC | TTCAGCAGCCGAGCCAATCA |
| MKRN3-2 | CTTTCTGGTCGGAAGATGGC | CACCTGCGGATACACCTAAT |
| MKRN3-3 | TGCAGCGTGGTATGGACAAG | AGAAGCACTGCCTCAACAGC |
| DLK1 exon1 | AACGTGTACCAAACGCTC | TGGGGGTCTCACACATC |
| DLK1 exon2+3 | GTCATTTCATTGMTGGGAAC | CAGGAATGAGGAAGTGAGGA |
| DLK1 exon4 | TCCCTAAACCBTCTTACTCC | ACAGACATCCCAGAAAATCC |
| DLK1 exon5 | CTGACTTTTCCTCTCCTMGC | TTGTTTTTGCATTGCGTTTG |

**Table 2. Clinical characteristics of patients with MKRN3 mutations.**

TS, Tanner Stage; BA, Bone Age, FSH b, basal FSH; LHb, basal LH; LHp, peak LH; UL, Uterine Length; TV, Testicle Volume.

| *MKRN3* mutation | Familial | Age at puberty onset (years) | Age at diagnosis (years) | TS | BA (years) | LHb (IU/L) | LHp (IU/L) | Peak LH/FSH ratio | E2 (pg/ml) | T (ng/dl) | UL (mm) | TV (ml) |
| --- | --- | --- | --- | --- | --- | --- | --- | --- | --- | --- | --- | --- |
| p.Leu474Met | Yes | 9 | 9.3 | 2 | 12.5 | 0.32 | 9.5 | 0.73 | 17.8 | 56.8 | —— | 4 |
| p.Ile357Met | Yes | 7.2 | 7.3 | 2 | 9 | 1.52 | 9.7 | 1.1 | 48.9 | 30.9 | 19 | —— |
| p.Glu380Lys | Yes | 7.8 | 8.3 | 3 | 11.1 | 0.94 | 5.4 | 0.72 | 53.3 | 18.8 | 25 | —— |
| p.Leu225Val, p.Glu380Lys | Yes | 7.5 | 8.2 | 3 | 11.2 | 2.31 | 97.1 | 2.68 | 59.6 | 40.7 | 23 | —— |
| p.Glu380Lys | Yes | 7.8 | 7.9 | 2 | 9.2 | 0.07 | 16 | 1.26 | 17.6 | 19.3 | 20 | —— |
